# Parametric Finite Element Model and Mechanical Characterisation of Electrospun Materials for Biomedical Applications

**DOI:** 10.3390/ma14020278

**Published:** 2021-01-07

**Authors:** Katarzyna Polak-Kraśna, Emilia Mazgajczyk, Pirjo Heikkilä, Anthimos Georgiadis

**Affiliations:** 1 Biomechanics Research Centre, National University of Ireland, H91 TK33 Galway, Ireland; 2 Institute of Product and Process Innovation, Leuphana University Lüneburg, 21339 Lower Saxony, Germany; georgiadis@uni.leuphana.de; 3 Faculty of Mechanical Engineering, Centre of Advanced Manufacturing Technologies–Fraunhofer Project Center (CAMT-FPC), Wroclaw University of Science and Technology, 50-370 Wrocław, Poland; emilia.mazgajczyk@pwr.edu.pl; 4 VTT Technical Research Centre of Finland Ltd., FI-02044 VTT Tampere, Finland; pirjo.heikkila@vtt.fi

**Keywords:** electrospinning, FE, tensile testing, nonwoven, modelling, poly(ethylene oxide) (PEO)

## Abstract

Electrospun materials, due to their unique properties, have found many applications in the biomedical field. Exploiting their porous nanofibrous structure, they are often used as scaffolds in tissue engineering which closely resemble a native cellular environment. The structural and mechanical properties of the substrates need to be carefully optimised to mimic cues used by the extracellular matrix to guide cells’ behaviour and improve existing scaffolds. Optimisation of these parameters is enabled by using the finite element model of electrospun structures proposed in this study. First, a fully parametric three-dimensional microscopic model of electrospun material with a random fibrous network was developed. Experimental results were obtained by testing electrospun poly(ethylene) oxide materials. Parameters of single fibres were determined by atomic force microscopy nanoindentations and used as input data for the model. The validation was performed by comparing model output data with tensile test results obtained for electrospun mats. We performed extensive analysis of model parameters correlations to understand the crucial factors and enable extrapolation of a simplified model. We found good agreement between the simulation and the experimental data. The proposed model is a potent tool in the optimisation of electrospun structures and scaffolds for enhanced regenerative therapies.

## 1. Introduction

Electrospun materials have unique properties, making them excellent candidates for biomedical applications such as tissue engineering [1,2,3], wound dressings [4,5,6,7,8] and drug delivery vehicles [9,10]. The large surface-to-volume ratio and high porosity combined with small pore sizes improve breathability, impenetrability to bacteria and contamination, as well as fluid absorptivity, which, combined with the incorporation of medicinal substances, enables the development of active wound dressing, accelerating the healing process [11,12].

Electrospinning is a technique developed at the beginning of the 20th century [13] to produce micro- and nanoscale fibres from a polymer solution or melt using electrostatic forces. It has been widely used to develop tissue engineering scaffolds supporting cell growth. It has been shown that the dimensions of the pores and fibres that can be achieved in electrospun scaffolds resemble a natural cell environment; the extracellular matrix (ECM) [5,6,14,15,16]. ECM is a natural in vitro scaffold, a 3D framework to which the cells attach and develop. ECM guides cells’ differentiation, proliferation, and growth by structural and mechanical cues [17]. Cellular behaviour can be modulated by scaffold properties such as porosity, fibre dimensions and mechanical properties. The introduction of stress onto the surface of a material has shown a positive effect on cells growth and proliferation [18,19,20,21]. Structural optimisation is vital for neural tissue engineering, where it is particularly important to provide directional guidance for nerve cell growth [19,22]. Ramakrishna and his group found that alignment of electrospun scaffolds increased the proliferation of nerve stem cells when compared to non-aligned structures [22]. Optimisation of these parameters will allow developing scaffolds with improved mechanical parameters to enhance regenerative therapies outcomes.

ECM also provides cells with informational signals via proteoglycans, glycosaminoglycans, and fibrous proteins (i.e., collagen, elastin or fibronectin) [23]. The capacity of co-spinning scaffolds with additional substances enables further functionalisation of a scaffold within the production process. Electrospun scaffolds made of or with the addition of natural polymers that further mimic the native tissue environment have been successfully applied in tissue engineering [5,7].

Electrospinning allows simultaneous polling of piezoelectric fibres during the production process, which makes it particularly interesting for bone and neural tissue engineering [24,25]. When polled, direct and reverse piezoelectric effects can be used to stimulate cell growth with electric and mechanical stimuli [26]. Understanding the mechanical stimulus’ magnitude and distribution in these structures would greatly improve scaffold optimisation capacities.

Electrospun scaffolds were also widely used for bone [8] and soft tissue engineering, in particular for vascular [3,14,27], tendon [11], ligament [28], and skeletal muscle [1]. Multiple materials can be used for electrospinning in medical applications, including synthetic biocompatible polymers and blends (poly(ethylene oxide) (PEO) [29], poly(L-lactide) (PLLA) [30], poly(ε-caprolactone) (PCL) [31,32], polyurethane (PU) [14], poly(ethylene glycol) (PEG) [27]), natural polymers (collagen [33], chitosan [34] or silk fibre [35]), and piezoelectric materials (poly(vinylidene fluoride-trifluoroethylene) (PVDF-TrFE) [24]). PEO is a water-soluble polymer that has been used as a model polymer in research due to its processability and biocompatibility. As it can be electrospun from water solution, it eliminates risks related to toxic solvent residues in the material [29,36,37].

Reasons for the increased interest of scientists in nanofibres during recent decades include their unique properties such as their huge surface to volume ratio and enhanced mechanical characteristics including a high tensile strength and Young’s modulus within such a small volume. This effect can be attributed to molecular alignment, an effect of very large effective spin draw ratio during the electrospinning process. This property results in mesoscopic anisotropy but relatively small crystallinity and thus unique mechanical properties [15,16,38].

Electrospinning is a straightforward method to produce nanofibres; however, the multitude of parameters influencing the behaviour of electrospun materials makes them difficult to optimise using experimental approaches [29]. Moreover, electrospinning is a slow process due to its low output rate which makes the optimisation of resulting materials very time-consuming. Development of tools enabling predicting the properties of electrospun materials would greatly enhance the development of optimal structures with desired parameters.

It was proposed that the structures used in tissue engineering should best reflect the properties of the ECM, including mechanical properties. The finite element (FE) method can be used to find the optimal parameters governing scaffold performance [39] such as porosity, fibrous alignment, and distribution of stress. FE method allows for scaffold design optimisation by predicting mechanical responses of cells and scaffolds under applied loads. It can potentially mimic the morphology of tissue using mesh elements, material properties, and physiological loads by applying loading conditions and constitutive stress-strain equations [40].

While multiple studies have reported FE models of scaffolds produced by various techniques, there are limited publications on modelling three-dimensional mats made by electrospinning [41,42]. Yin and Xiong presented a parametric microscopical FE model of electrospun mat consisting of single nanofibres assuming that a single layer yields results comparable to the entire structure [43]. Chung and Koh compared 2D and 3D models with cross-linked layers and reached a conclusion that a single layer gives satisfactory results in terms of fracture behaviour [44]. Existing models of materials with random fibrous distribution discussed in the works of Liao et al. [45] and Bais-Singh et al. [46] are based on the assumption that fabric consists of separate two-dimensional layers. Goutianos et al. developed a 3D model of cellulose nanopaper bonded with hydrogen bonding with fibres randomly distributed in all three directions [47]. In the work by Zündel et al., a 2.5D modelling approach was proposed where a 2D network of randomly distributed fibres is enriched with out-of-plane fibres interactions [48]. We propose that an additive process of electrospinning produces a far more interconnected structure than can be represented by combining separate layers. The interlaminar fibrous connections developed gradually within layers enable the structural stability of electrospun materials and should be accounted for. According to the authors’ knowledge, there are no existing 3D models representing a random fibrous network (RFN) typical for electrospinning process with randomised dimensional and mechanical properties of single fibres.

In this study, we developed a modelling tool that enables simulation of the mechanical behaviour of electrospun materials to facilitate and improve the design and optimisation of electrospun structures for different applications. Additionally, we performed optimisation of electrospinning parameters of poly(ethylene) oxide (PEO) to obtain high-quality single fibres and large-scale electrospun mats. Geometrical and mechanical characterisation of single electrospun fibres and electrospun mats was performed. Finally, we looked into the mechanisms contributing to electrospun material failure by analysing post-failure samples with scanning electron microscopy (SEM).

We developed a fully parametric microscopic finite elements model of electrospun material consisting of nanofibres. The electrospun random fibrous network was modelled with optional parameters, allowing for partial or full fibrous alignment. Adjustable parameters within the model are sample dimensions, the number of fibres, fibres’ alignment, geometrical and mechanical properties of single fibres and the ratio of fibrous connections within the sample. The structure of the electrospun mat and the fibre alignment were modelled based on scanning electron microscopy images obtained for electrospun PEO samples. Mechanical characteristics of single fibres were determined with the nanoindentation method inside an atomic force microscope (AFM). Tensile testing of electrospun PEO mats was performed to provide validation data for the model not previously reported in the literature.

Due to computational limitations, solving of a model consisting of up to five thousand fibres was possible. We propose an extrapolation method that enables solving a simplified model which can be extrapolated to a full-scale model based on multiple simulations and analysis of correlations between input parameters and resulting material behaviour that we performed. The extrapolated model results were then compared with the experimental results of tensile tests to validate the modelling and extrapolation output.

## 2. Materials and Methods

### 2.1. Finite Elements Model

The model of electrospun nonwoven nanofibrous fabric was developed with FE method in an ANSYS environment. The model was built using ANSYS Parametric Design Language (APDL) in ANSYS Mechanical APDL (Ansys, Inc., Canonsburg, PA, USA) package.

The fibrous elements were modelled as trusses; due to the very small diameters of fibres in comparison to their lengths, their bending energy was assumed negligible. The starting and ending points of trusses were distributed randomly within given coordinates range in order to reproduce the random nature of the electrospun fibrous network. The alignment of fibres could be controlled by adjusting these ranges to achieve partially or entirely aligned fibres in different directions. The fibres were modelled gradually increasing their location in the *z* direction to mimic the structure resulting from additive process formed of interconnecting layers. A three-dimensional structure was thus formed. The thickness and parameters determining the number of layers, as well as the number of fibres within one layer, can be adjusted. After the process of randomly distributing the fibres, a rectangular sample was selected with dimensions according to experimental protocol. Fibres that were not attached to sample edges or other fibres were removed from the structure to avoid under constraining of the model. The geometrical and mechanical parameters of fibres, i.e., the magnitude of thickness and Young’s modulus, were applied to fibres randomly within a user-specified range. This was done to accurately mimic the electrospun material’s characteristics; it was shown in the experimental part of this study that the properties of fibres within a single electrospun structure can vary significantly. Additionally, the mechanical properties of fibres reported in the literature tend to have high standard deviations [49,50,51,52,53], which was represented in the model by randomised assignment of parameters amongst fibres. Additional parameters influencing the mechanical behaviour of the structure were the connections formed at the interface between the fibres. The presence of these connections may vary significantly depending on the polymeric solution concentration, electrospinning process parameters and material post-processing such as point-bonding or cross-linking. The material considered in this study did not undergo postprocessing. Structural characterisation in the experimental part of this study showed some fibres to be interconnected, whereas others were not. Part of the model constituent fibres were therefore bonded together (using bonded contact pair type in ANSYS where no sliding and no separation between bodies is allowed), and others were set to non-contact pairs. The number of these connections is another parameter that can be adjusted in the model; the user can determine how many fibres are bonded at their cross-sections and how many are allowed to slide against each other.

Discretisation of the model was realised by dividing the trusses into elements at their intersections. A truss element with uniaxial tension–compression behaviour and three degrees of freedom at each node was chosen (LINK180). Fibre diameters were assigned in the range 200–300 nm. A preload in range −20–20% strain was applied to random fibres to account for different initial tension in fibres observed in microscopical analysis. The SEM images obtained showed slight curvature of part of the fibres, which plays an important role in the mechanical response of the materials [48]. In the initial loading phase, this would delay the onset of load bearing by those fibres. This parameter was accounted for by applying negative strain (−20–0%) which effectively introduced gradual onset of load bearing in fibres within the material. The material parameters used in experimental study were applied based on experimental results with Young’s modulus ranging 0.2–8 GPa (with different sub-ranges applied in particular simulations) and Poisson’s ratio 0.3.

The uniaxial tensile test was simulated by constraining the modelled sample on the left edge where all degrees of freedom were taken away. The displacement was applied at the right edge, where a rigid extension was added to ensure uniform distribution of applied strain. The solver parameters were set to nonlinear analysis, large deflections, and load stepping.

Over 20 simulations with different input parameters were performed to determine the dependencies between parameters and understand their influence on model’s behaviour. The five input parameters were variables: number of fibres, connections ratio, sample thickness, and range of single-fibres Young’s modulus. The next step was to graph the above dependencies and analyse their correlations. This analysis allowed for the extrapolation of the model to a model with a larger number of fibres. The parameters used for extrapolation of the model were the number of fibres and ratio of connections.

### 2.2. Electrospinning

Polymer solution used in the process was 4 wt% PEO with molecular weight of 900,000 g/mol (Sigma Aldrich, St. Louis, MO, USA) in distilled water. It was prepared by adding 4 g of PEO to 96 g of distilled water and shaking vigorously for few minutes to achieve homogenous solution. Then the solution was left for four hours before using it for electrospinning to ensure all bubbles had evaporated.

The electrospinning equipment used for the development of single fibres was a vertical setup consisting of a high voltage generator, a syringe pump, a rubber tube connecting the pump with a nozzle, and a flat grounded collector. A blunt needle with a diameter of 0.4 mm was used as a nozzle.

Electrospinning of single fibres was performed during one day in order to ensure stable environment conditions and to exclude the influence of temperature and humidity on experimental results. The mean value of temperature measured repeatedly during the experiments was 19.3 °C (± 0.1 °C) and the atmospheric pressure remained at the level of 1014 hPa during all experiments. The following parameters were optimised for fibre quality and set as constant during the process; nozzle-collector distance (22 cm), duration of electrospinning process (3 min), and feed rate describing the speed of the solution being pushed from the nozzle (0.016 mL/min). Voltage was used as a variable in the range of 10–14 kV. Fibres were collected on a glass slide to enable microscopical imaging.

Analysis of quality of samples and geometry of fibres was performed using a Confocal Laser Scanning Microscope (CLSM, LSM 700, Carl Zeiss AG, Oberkochen, Germany). Samples were examined in terms of fibrous density and number of drops and beads.

Samples for tensile tests were developed with a horizontal electrospinning setup with a rotating mandrel allowing to obtain a homogenous sheet of electrospun material 18 cm × 30 cm. In order to achieve optimal electrospinning performance, defined by the efficiency of fibre production, a lack of beads or wet spatters, and homogenous fibres, parameter optimisation was performed. Parameters assessed in the optimisation process were the concentration of the polymer solution (2–4 wt%), voltage (8–30 kV), and nozzle–collector distance (10–20 cm). A series of short-time electrospinning processes (3–20 min) were performed. The optimised input parameters were a needle–collector distance of 20 cm, a solution feeding rate of 0.3 mL/h, and a voltage of 20 kV. The electrospun mat was produced within 9 h. The average temperature was 22.1 ± 0.3 °C, and a relative humidity of 37% was maintained throughout the process.

The obtained fibrous mat was assessed quantitatively by measuring the thickness of the electrospun layer and qualitatively with a scanning electron microscope. For SEM analysis, samples were mounted on sample holders and coated with thin layer of gold (Balzers SCD 050 Sputter Coater, Baltec AG, Pfäffikon, Germany). SEM imaging (JEOL JSM-6360LV, Jeol Ltd., Tokyo, Japan) was performed with a series of magnifications (100–20,000×); the images were used to measure fibre diameters and their standard deviations using ImageJ software (v.1.51k, National Institutes of Health, Bethesda, MD, USA).

### 2.3. Single Fibres Characterisation

Fibre thickness distribution was measured using CLSM and the quality of fibres was assessed by the number of beads—droplets formed along the wires during electrospinning process.

**Figure 1:**
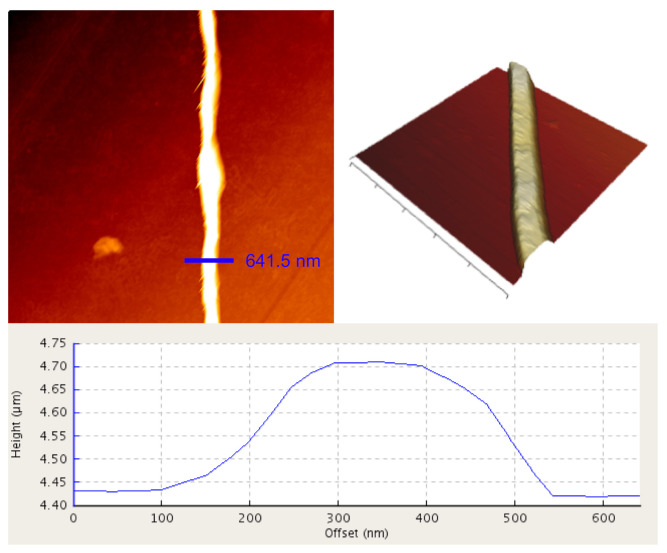
One of the fibres imaged in an atomic force microscope (**left**) with its cross-section plot (**bottom**) and 3D model (**right**).

The elastic modulus of single fibres was measured using nanoindentation in AFM (JPK NanoWizard II, Bruker Nano GmbH, Berlin, Germany) in force mode. The indenter used for all experiments was four-sided pyramid with spring constant 0.292 N/m and the operating frequency 66 kHz (HYDRA 6 V-100 NG from JPK, Bruker Nano GmbH, Berlin, Germany). Each fibre was initially imaged in tapping mode to determine the optimal position for nanoindentation experiment. For every experiment, the cross-sectional area of the fibre was plotted (Figure 1). Only measurements complying with the Hertzian assumption for half-space bodies contact were included in data analysis. During nanoindentation procedure in force mode, the indenter was being pushed against a fibre and reaction force was recorded. Force–displacement curves were plotted. In order to determine the values of Young’s modulus from sample loading curve, Hertz model was applied, according to the methodology previously developed in our group [53]. Indentations did not exceed 10% of fibrous height to ensure requirements for the model applications were met. Indentation model for four-sided pyramid indenter used in the experiment [53] is expressed by

(1) $$F=\frac{E_{s}}{1-\mu^{2}}\frac{\tan\alpha }{\sqrt{2}}\delta^{2},$$

where *F* is the indentation force, *E_s_* is Young’s modulus of a sample, *μ* is Poisson’s ratio of a sample, *α* is the face angle of the pyramid indenter, and *δ* is the indentation depth. Nanoindentations were performed for five fibres within each sample. 8 to 12 force responses were measured within one fibre and an average value was calculated.

### 2.4. Experiments for the Validation of the Model

For experimental validation of the model, tensile testing of electrospun samples was conducted and the results were compared with simulated uniaxial tensile test results from the model.

**Figure 2:**
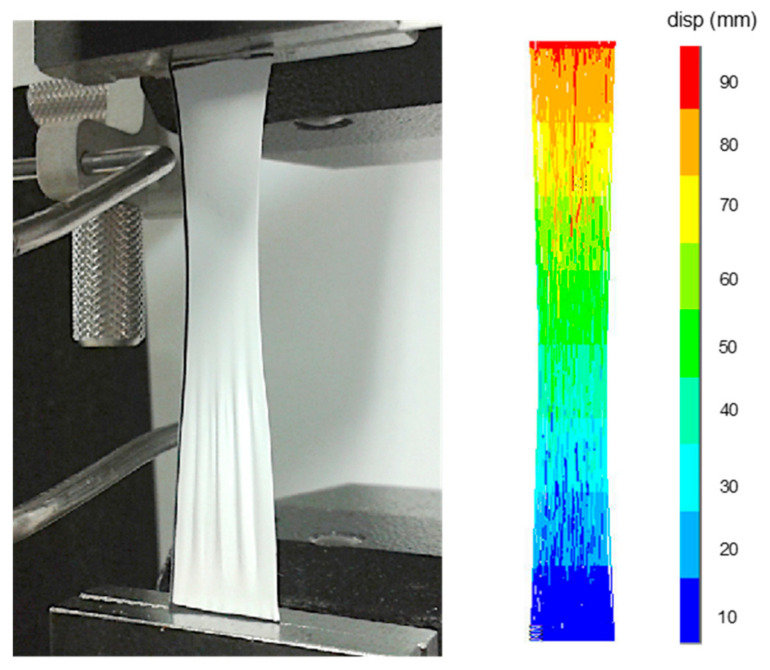
Sample clamped in the jaws of tensile testing machine during uniaxial tensile test (**left**). Deformed shape of the electrospun structure FE model in tension under displacement 100 mm (**right**).

Equally sized samples with the width of 1.25 cm and gauge length 5 cm (¼ of the norm for nonwovens testing [54]) were cut out of the electrospun mat. The thickness of every sample was measured using a digital micrometer (Helios-Preisser GmbH, Gammertingen, Germany) with measuring range of 0–75 mm and accuracy ± 0.007 mm. The thickness was measured five times within one sample and the average value was calculated. Samples were subjected to uniaxial tensile test with tensile testing machine (Universal Testing System Instron 4505, Instron, Norwood, MA, USA) equipped with 1 kN load cell. The experiment was performed with a quasi-static speed of 2 mm/min to simulate the static loading the material is subjected to in medical applications. Adhesive tape was attached to each specimen to improve grip in tensile testing machine clamps. A sample in testing grips subjected to a tensile test is presented in Figure 2. Force–displacement values were recorded and stress–strain curves were plotted from which Young’s modulus, ultimate stress and strain to failure were evaluated.

Samples were analysed during and after the tensile test with SEM in order to understand the mechanisms of the material’s failure. To enable the analysis during the tensile test, the experiment was stopped after the sample exceeded yield stress and a piece of electrospun fabric was prepared for imaging. To maintain the stress state achieved during testing, the fibres were not allowed to go back to their relaxed state but fixed on sample holders in their stressed state. Samples tested after the material’s rupture were imaged after relaxation.

## 3. Results

### 3.1. Simulations Results

The solution and results were obtained from more than twenty simulations with varying input parameters. The effect of the number of fibres, connections ratio, sample thickness, and the range of single-fibre Young’s modulus were determined. The range of Young’s modulus applied to single fibres had a linear influence on the increase in the maximum stress value in the model. With increasing the thickness of a sample and thus decreasing the fibrous density, the maximum stress decreased. The number of nodes was related to the number of fibres in the model, the thickness of the sample (and thus fibres density) and connections ratio. The increase in node number exponentially decreased the resulting magnitude of strain. The results obtained within the model could be extrapolated to real case sample by using observed dependencies.

The deformed shape of the model after applying 100 mm displacement is represented in Figure 2. This reveals the subtle effect of necking. The deformation was observed in the entire sample and within single fibres on the micro-level of fibrous structure, which can be seen as single blue and red fibres along the structure. 

**Figure 3:**
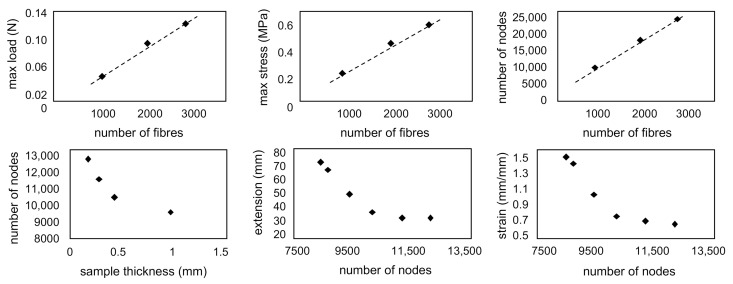
Dependencies of parameters observed in the model showing (**top**) the influence of the number of fibres on the maximum load and stress and the number of nodes, (**bottom**) the sample thickness on the number of nodes, and the number of nodes on extension and strain in the model.

Analysis of parameters’ dependencies based on over 20 simulations with different input parameters have shown that the number of nodes (connections) in the model increased exponentially with increasing fibre density and increased linearly with increasing the number of fibres. An increase in the number of fibres increased the maximum loads in the structure linearly, and hence the stress. The number of connections decreased the resulting strains significantly. Some of the most relevant dependencies are presented in Figure 3. 

Extrapolation of the results was performed based on data obtained from model with Young’s modulus of single fibres in the range of 4–5 GPa, consisting of 1100 fibres resulting in 759 nodal connections in the model. The sample dimensions were based on experimental results (thickness 0.019 mm, width 12.5 mm, length 50 mm). Values obtained directly from the model showed a maximum force equal to 0.08 N, a maximum stress of 0.35 MPa, an extension at maximum load of 48 mm, a strain at maximum stress of 0.96 mm/mm and Young’s modulus of the entire structure equalling 1.02 MPa.

The calibration of the model aimed at obtaining values in the range of experimental stress, strain, and Young’s modulus of the structure by adjusting free parameters: number of fibres and ratio of connections. The model was extrapolated to 9000 fibres with a connections ratio of 25%, resulting in 1863 nodes. The resulting mechanical parameters after extrapolation were a maximum stress of 1.54 MPa, a strain at maximum stress of 0.17 mm/mm, and a Young’s modulus of the entire structure equalling 22.13 MPa. 

### 3.2. Electrospinning and Single Fibres Properties

Successful electrospinning was obtained with voltages between 11 and 13 kV. Below and above that range of voltage, no fibres were formed on the glass slide. The highest density of fibres was obtained with voltage 11 kV, but it also resulted in the highest amount of beads and solution drops that decreased the quality of fibres significantly. The number of beads was minimal in the samples electrospun with 13 kV voltage; when compared with samples obtained with 11 kV, only 11% of beads were present on the surface of fibres at 13 kV. With higher voltages, the number of beads decreased in general. We did not observe a clear dependency between the voltage and diameter of fibres; however, the highest average diameters, 520 ± 70 nm, resulted from voltage 11 kV, whereas the lowest, 390 ± 40 nm, resulted from voltage 12 kV. The overall average fibre diameter obtained in the whole voltage range was 440 ± 90 nm.

The Young’s modulus obtained with AFM nanoindentations showed high standard deviations, both within single fibres and in different samples. We obtained an average Young’s modulus of 4.1 ± 2.6 GPa, with the lowest value being 0.9 GPa and the highest being 10.8 GPa. Average values for all fibres are presented in Table 1. We did not determine a dependency between electrospinning voltage and Young’s modulus. Whether the fibres had beads on them did not seem to influence their elasticity.

### 3.3. Electrospinning and Characterisation of Electrospun Material

**Figure 4:**
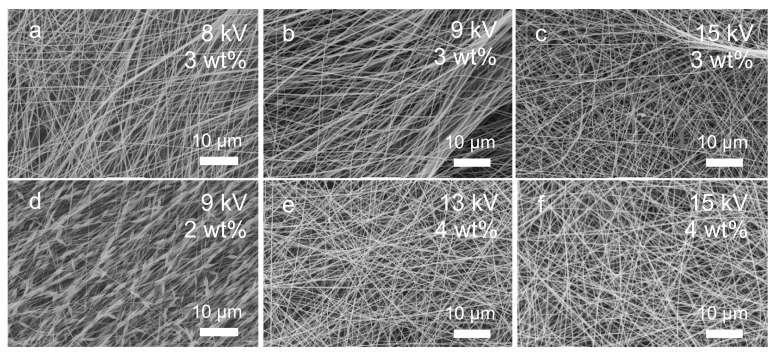
Comparison of resulting electrospun structures with different poly(ethylene oxide) concentrations and different voltages. All images were obtained with magnification 2000×. Electrospun mats obtained with (**a**) 8 kV voltage and solution concentration 3 wt%, (**b**) 9 kV voltage and 3 wt% solution concentration, (**c**) 15 kV voltage and 3 wt% solution concentration, (**d**) 9 kV voltage and 2 wt% solution concentration, (**e**) 13 kV and 4 wt% solution concentration, (**f**) 15 kV voltage and 4 wt% solution concentration.

Optimisation of the electrospinning parameters to produce large-scale mats was performed by assessing the density of fibres and appearance of drops and beads. The optimal setup resulted in a high density of fibres, with no drops or beads. The resulting fibre meshes, as observed in SEM, are presented in Figure 4 along with the parameters used in the process. Lower PEO concentrations resulted in lower densities of fibres, and there was a clear trend showing an increase in fibres density in samples prepared with 2, 3, and 4 wt%, with the latter producing the maximum densities observed. Electrospinning with 2 wt% consistently produced the “pearl-on-the-string” type of structure with multiple beads (Figure 4). The number of fibres was also dependant on voltage; no fibres were formed below 8 kV and above 20 kV. The optimal distance was chosen as 15 cm due to the large area of fibre collection and the minimum contamination by drops or solution spatters, and the voltage that yielded the best results was 15 kV for 4 wt% solution. This was later increased to 20 kV at a 20 cm nozzle–collector distance to maintain electric field density. A longer distance allowed for collection of a larger electrospun sheet. The optimal solution feeding rate was 0.3 mL/h.

**Figure 5:**
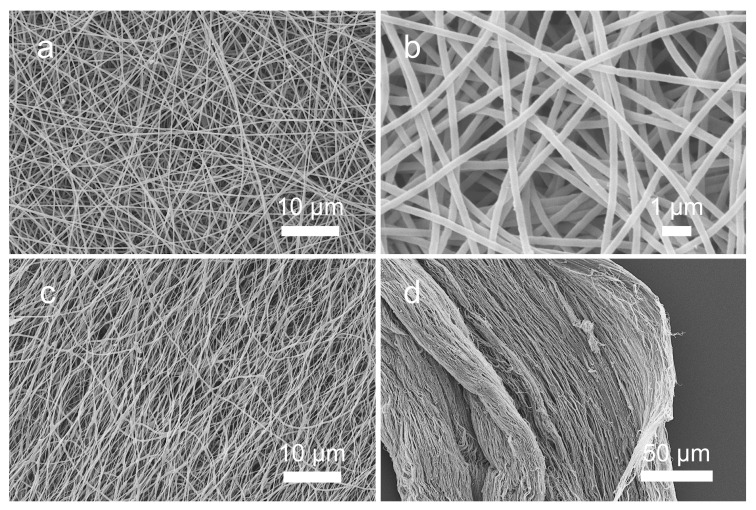
(**Top**) Appearance of electrospun nanofibrous nonwoven sheet of PEO obtained with SEM, magnification 2000× (**a**) and 10,000× (**b**). Regular structure of fibres is visible as well as fibrous rough edges of single fibres. (**Bottom**) The same electrospun sample after rupture in tensile test shown in the middle region at 2000× magnification (**c**) and at the edge with 500× magnification (**d**).

The electrospun mat intended for tensile testing was produced within 9 h. The average temperature was 22.1 ± 0.3 °C, and a relative humidity of 37% was maintained. Analysis with SEM revealed that the long electrospun mat had a homogenous distribution of fibres (Figure 5). Some of fibres were straight and other ones slightly curvy. There was some roughness visible on the edges of part of the fibres. There were no defects on single fibre’s surfaces visible under the highest microscope magnifications. The diameters were in the range between 238 and 354 nm, with an average value of 298 nm and a standard deviation of 20 nm.

**Figure 6:**
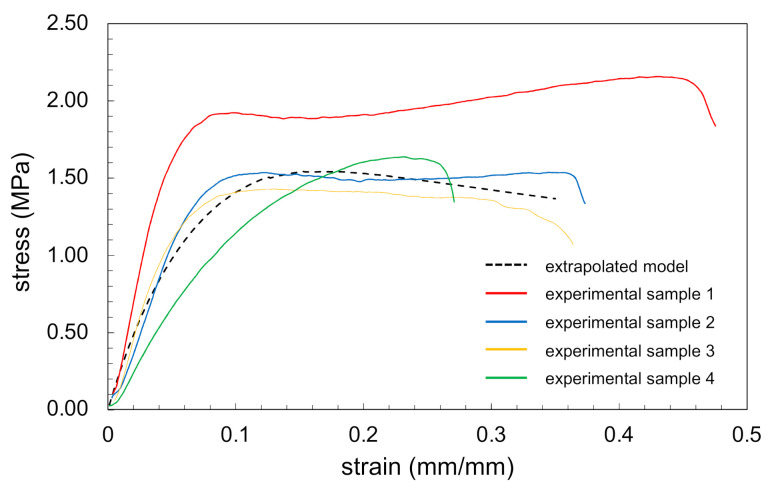
Stress–strain curves obtained in tensile tests of electrospun samples obtained with 4 wt% PEO solution with 20 kV, 20 cm nozzle-collector distance, 0.3 mL/h feed rate, on a rotating mandrel (solid curves), and extrapolated from the FE model (dashed curve).

Tensile tests were performed on four electrospun PEO samples with an average thickness of 0.019 mm. The stress–strain curves obtained for all samples are shown in Figure 6. Almost all curves had shapes typical for ductile polymer samples, characterised by two maximums and a ductile plateau where necking was observed. This type of plot is also typical for fibrous structures where fibres are gradually subjected to loading and gradually fail. Failure of samples took place at an average stress of 1.75 ± 0.27 MPa, with values ranging between 1.5 and 2.2 MPa. The average ultimate load observed was 0.40 ± 0.05 N and, surprisingly, this result was obtained in a sample with lowest thickness, but we did not observe a clear dependency between thickness and ultimate load values. The maximum load overall was in the range 0.33–0.45 N. Extensions for these loads were equal to 7.5 and 21.3 mm, respectively. Strain reached on average 30%, ranging between 15% and 42% of the sample’s initial length. Additionally, the values for the first maximum point were determined to enable comparison with the FE data where stress reached on average 1.31 ± 0.65 MPa (in range 0.23–1.96 MPa) and strain corresponding to that stress was on average 0.15 ± 0.05 mm/mm (in range 0.10–0.23 mm/mm) (Table 2). The stress–strain plot obtained from the model after extrapolation is shown in Figure 6 as a dashed curve.

Post tensile analysis of ruptured samples with SEM exemplified the mechanisms of damage in the samples that took place beyond the yield stress limit (Figure 5, bottom right). In the figure, the edge of the ruptured area of the sample is presented with broken fibres clearly visible. Fibres went back to a relaxed state after maximum stress but are visibly oriented in the stretching direction.

In Figure 5 (left), comparison of the same PEO sheet before and after failure in the tensile test is shown. The area of electrospun material presented in the figure is remote from the ruptured edge and the visible fibres did not directly undergo failure but were subjected to a long-distance effect. Fibres before the test were almost perfectly straight and homogenous in the diameter, whereas after rupture we observed curling of the fibres and diameter’s shrinkage resulting from plastic deformations during stretching.

A comparison of tensile testing data results and extrapolated model results for 9000 fibres is presented in Table 2. The average thickness of samples subjected to tensile testing was 0.019 mm. As the model did not capture plastic deformation, the plot obtained from the model only shows the first peak of stress present in the experimental stress–strain curves, and these values were compared to show that the model calibration was accurate and validate the FE results. Extrapolated stress reached 1.54 MPa, which was slightly higher than the average stress at that point obtained experimentally but within the standard deviation and inside the range of values obtained experimentally, between 0.23 and 1.96 MPa. The strain achieved for this level of stress in the extrapolated model was 0.17 mm/mm, which was near the experimental average equal to 0.17 mm/mm and within the experimental range, 0.10–0.23 mm/mm. The Young’s modulus of the entire structure calculated from the model was 22.13 MPa, which was slightly lower than the experimental average of 28 ± 11 MPa, but again within the standard deviation and experimental range of 14–44 MPa.

## 4. Discussion

The model presented in this work is a first attempt to recreate the material obtained within the gradual electrospinning process represented by microscopic fibrous structure with random fibre distribution and fibre properties randomisation. The model enables a high level of parametrisation including sample dimensions, fibrous alignment, fibre diameter and Young’s modulus range, the number of layers, density of fibres within a layer and the ratio of fibrous connections. We present extensive analysis of parameter dependencies based on a high volume of simulation data. This high-level parametrisation enables applying the proposed model to multiple scenarios also beyond electrospinning for structures such as nonwoven textiles or soft tissue consisting of collagen fibres [55].

Existing models of electrospun structures propose 2D solutions [41,42,44,56] or consist of separate two-dimensional layers [45,46]. We propose that the nature of interpenetrating layers resulting from the electrospinning process should be assessed with a 3D approach to account for interlayer interactions between fibres. Additionally, we demonstrate the first model where an RFN is represented with its intrinsic random distribution of geometrical and mechanical properties.

Due to high complexity of the model, we propose that the results obtained in a model with a limited number of fibres can be extrapolated to real-size sample based on analysis of parametric correlations we performed. This enables the employment of the model when limited computational resources are available. However, due to the parametric nature of the model, a full-scale computational analysis can be performed. The extrapolation was performed using results from the simplified model by adjusting the number of fibres and fibrous connections and calculating the resulting stress–strain data based on parameter correlation. This approach was validated by comparing the extrapolated data with experimental tensile results of electrospun samples. The results show good agreement.

Analysis of parameters’ dependencies showed that the number of nodes (connections) in the model increased exponentially with an increasing fibre density and increased linearly with an increasing number of fibres. The increase in number of fibres increased linearly the maximum loads in the structure and hence the stress. Connections between fibres in electrospun material seemed to be one of the crucial parameters that influenced stiffness and extensibility of obtained structures. The number of connections decreased the resulting strains significantly. Similar findings were presented in the paper by Goutianos et al., where the Young’s modulus was predominantly determined by density and the strength of connections [47]. Based on these dependencies, an extrapolation to a real-size model and its validation was possible.

Optimisation of electrospinning process parameters to obtain a high quality of fibres and mats was performed for two different electrospinning setups; a vertical setup with a flat collector used to obtain single fibres, and a horizontal setup with a rotating mandrel to produce large mats. We found that lower concentration (2 wt%) of PEO solution resulted in structures with multiple beads which were not present when using concentrations of 3 and 4 wt%, whereas 4 wt% produced higher densities of fibres.

Moreover, determination of mechanical parameters of PEO single fibres and electrospun mat was performed. Single fibre characterisation was completed on a large group of samples (20 fibres and 200 nanoindentations performed) to enable reliable statistical distribution of data. The method we employed was nanoindentation with AFM. There is limited literature available on using this technique for electrospun fibres characterisation [57]. The average Young’s moduli obtained for single fibres in this work (4.1 ± 2.6 GPa) were slightly lower than those obtained in the study of Bellan et al. (7.0 GPa) [52], who used piezo-driven mechanical oscillations of individual suspended fibres to measure mechanical properties. This seems to be a typical feature of electrospun samples to show a wide range of mechanical properties and high standard deviations common in the literature reports [49,50,51]. Electrospinning itself is a highly random process producing a range of different outcomes even during one experiment, which can lead to inhomogeneity in samples and even single fibres in terms of their shape, diameter and quality. We observed inhomogeneities of single fibres such as beads and rough edges which may significantly influence mechanical testing results, particularly in structures with sizes measured in range of hundreds of nanometres. AFM is a very sensitive technique and can be susceptible to surface artifacts. Moreover, parameters such as the angle of an indenter tip can have a massive influence on results.

The tensile properties of electrospun mats have also been shown to lie within a wide range of values in terms of ultimate stress (0.23–1.96 MPa), strain to failure (0.10–0.23 mm/mm) and Young’s modulus (14–44 MPa). This was in agreement with previously reported literature ranges [58]. These large variations in parameters are inherent features of electrospun structures and have to be accounted for in models. They were reflected in the presented FE model by introducing a random assignment of mechanical properties to single fibres within a selected range. Analysis of post-failure samples showed that fibres fail gradually, which explains the shape of the stress–strain curves obtained during testing with a long plateau following the yield point. After failure, we observed significant deformations of single fibres and high curling ratio, which points to a significant spring response after a fibre’s rupture.

Comparison of extrapolated and experimental results has shown high compatibility. The number of fibres and connections ratio were used to extrapolate the model. Due to the lack of existing data on the number of connections existing in electrospun structure and their strength [56], the connections ratio and fibre number were each used as calibration parameters. The model could be further validated by providing experimental results, helping us to understand the nature of how connections between fibres influence the overall behaviour of electrospun mats. The proposed model assumed linear-elastic behaviour of single fibres based on small strains obtained experimentally, whereas additional mechanical parameters would allow for incorporating more sophisticated materials models describing hyperelastic and plastic nature of deformation. The curvature of fibres was accounted for by applying negative strains of the fibres in the model; further analysis with the introduction of bending could be implemented to assess the efficacy of this approach.

The crucial factor ensuring proper extrapolation was understanding the dependencies between various parameters in the model. We have performed multiple simulations to describe these dependencies. Multiparameter analysis could be expanded to develop a correlation matrix and propose a tool to perform extrapolation automatically. This would be practical for wider applications, particularly when introducing additional parameters, such as plasticity, into the model.

The proposed model is a potent tool to aid the optimisation of electrospun materials such as scaffolds for tissue engineering where optimal structure, porosity, fibrous alignment, and distribution of stress are crucial to mimic a native cell environment, the ECM, and guide cellular behaviour to enhance regenerative therapies in the future.

## Figures and Tables

**Table 1:** Young’s modulus (E) measured with AFM nanoindentations on single electrospun fibres with standard deviations (SD) and average (AV) Young’s modulus obtained for each electrospinning voltage.

Sample	Fibre	E (GPa)	SD (GPa)	Sample	Fibre	E (GPa)	SD (GPa)
11 kV	1	10.8	6.7	13 kV	1	4.8	5.0
	2	3.9	2.8		2	5.3	2.7
	3	4.2	2.4		3	2.2	0.9
	4	3.7	2.1		4	2.5	1.0
	5	2.4	1.4		5	2.1	1.7
	AV	5.0	3.0		6	1.2	1.1
12 kV	1	4.6	2.7		7	2.5	1.1
	2	3.7	2.0		8	3.0	0.7
	3	3.4	5.1		9	1.8	1.5
	4	8.4	11.4		10	0.9	0.9
	5	9.0	6.7		AV	2.6	1.3
	AV	5.8	2.4	Average		4.1	2.6

**Table 2:** Comparison of experimental, model and extrapolated mechanical properties of electrospun structure.

Data Type	Stress (1st Max)	Strain (at 1st Max Stress)	Young’s Modulus
	(MPa)	(mm/mm)	(MPa)
values extracted from model (1100 fibres)	0.35	0.96	1.02
extrapolated model values (9000 fibres)	1.54	0.17	22.13
experimental average values	1.31 ± 0.65	0.15 ± 0.05	28 ± 11
experimental range of values	0.23–1.96	0.10–0.23	14–44

## Data Availability

APDL code for ANSYS Mechanical APDL model presented in this study is openly available via GitHub repository Electrospun-APDL-model under the following link: https://github.com/polak-krasna/Electrospun-APDL-model.
